# *C/EBPβ* Promotes *STAT3* Expression and Affects Cell Apoptosis and Proliferation in Porcine Ovarian Granulosa Cells

**DOI:** 10.3390/genes9060295

**Published:** 2018-06-13

**Authors:** Xiaolong Yuan, Xiaofeng Zhou, Yingting He, Yuyi Zhong, Ailing Zhang, Zhe Zhang, Hao Zhang, Jiaqi Li

**Affiliations:** 1 Guangdong Provincial Key Lab of Agro-Animal Genomics and Molecular Breeding, National Engineering Research Centre for Breeding Swine Industry, College of Animal Science, South China Agricultural University, Guangzhou 510642, China; yxl@scau.edu.cn (X.Y.); 13424457731@163.com (X.Z.); 15521059247@163.com (Y.H.); yyzhong04@163.com (Y.Z.); zhezhang@scau.edu.cn (Z.Z.); zhanghao@scau.edu.cn (H.Z.); 2 College of Biological and Food Engineering, Guangdong University of Education, Guangzhou 510303, China; zhangmeixial@163.com

**Keywords:** ovarian granulosa cells, *C/EBPβ*, *STAT3*, cell proliferation, cell apoptosis

## Abstract

Previous studies suggest that signal transducer and activator of transcription 3 *(STAT3)* and CCAAT/enhancer binding protein beta *(C/EBPβ)* play an essential role in ovarian granulosa cells (GCs) for mammalian follicular development. Several C/EBPβ putative binding sites were previously predicted on the *STAT3* promoter in mammals. However, the molecular regulation of *C/EBPβ* on *STAT3* and their effects on cell proliferation and apoptosis remain virtually unexplored in GCs. Using porcine GCs as a model, the 5′-deletion, luciferase report assay, mutation, chromatin immunoprecipitation, Annexin-V/PI staining and EdU assays were applied to investigate the molecular mechanism for *C/EBPβ* regulating the expression of *STAT3* and their effects on the cell proliferation and apoptosis ability. We found that over and interfering with the expression of *C/EBPβ* significantly increased and decreased the messenger RNA (mRNA) and protein levels of *STAT3*, respectively. The dual luciferase reporter assay showed that C/EBPβ directly bound at −1397/−1387 of *STAT3* to positively regulate the mRNA and protein expressions of *STAT3*. Both *C/EBPβ* and *STAT3* were observed to inhibit cell apoptosis and promote cell proliferation. Furthermore, *C/EBPβ* might enhance the antiapoptotic and pro-proliferative effects of *STAT3*. These results would be of great insight in further exploring the molecular mechanism of *C/EBPβ* and *STAT3* on the function of GCs and the development of ovarian follicles in mammals.

## 1. Introduction

Ovarian dysfunction causes the reproductive failure and infertility in female mammals. The main functions of ovaries are to produce mature oocytes that can propagate the species [1,2] and synthetize the steroids that support secondary sexual characteristics [3,4]. It was widely thought that granulosa cells (GCs) supported the development and maturation of follicles though the complex interactions [5,6], and that the growth and proliferation of GCs play critical roles in the biological processes of recruitment, selection, atresia, ovulation and luteolysis of follicles [7,8]. Previous studies have found that steroids, growth factors, and cytokine factors secreted by GCs are essential for the survival and growth of follicles [9,10], and moreover, the high apoptosis of GCs can impair folliculogenesis and result in the increased follicular atresia [11,12].

In mammals, the signal transducer and activator of transcription 3 (*STAT3*) protein has been suggested to be involved in folliculogenesis [13,14]. It has been reported that *STAT3* expressed highly in porcine GCs [15], and porcine complementary DNA (cDNA) of *STAT3* is 93% and 90% homologous to humans and mice, respectively [15]. In mares, mRNA of *STAT3* expresses higher in adult ovaries than in fetal ovaries [13]. In chickens, phosphorylated *STAT3* is activated by the epidermal growth factor [15], which is known to decrease the *P450scc* and follicle-stimulating hormone receptor mRNA abundance to regulate the biological functions of GCs [16]. In mouse, reduced expression of *STAT3* enhances the early apoptosis rate of mGCs [17], and moreover, specifically deleted *STAT3* in ovarian GCs can impair fertility with significantly fewer litters and smaller litter size [18]. However, the cell function of *STAT3* is seldom investigated in porcine GCs.

Much evidence has suggested that CCAAT/enhancer binding protein beta (*C/EBPβ*) plays an essential role in female reproduction [19,20]. In mice and rats, *C/EBPβ* mRNA is specifically and rapidly induced by luteinizing hormone in GCs [20,21]. A targeted deletion of *C/EBPβ* results in reproductive defects in female mice [20]. Moreover, the GC-specific *C/EBPβ* knockout causes subfertility with the absence of corpus luteums in 70% and the reduction expression of *Ptgs2*, *Star*, and *Cyp11a1* in mice [19]. These results supported the proposed and essential role of *C/EBPβ* in GCs for mammalian folliculogenesis. However, the functions of *C/EBPβ* on cell apoptosis and proliferation remained virtually unexplored for ovarian GCs.

Previous studies report that *C/EBPβ* has a *cis*-acting element in the promoter *PGS-2* in rat GCs [21], and moreover, we found several *C/EBPβ* potential binding sites were predicted on the *STAT3* promoter of humans, mice, and pigs (see Materials and Methods). We hypothesized that *C/EBPβ* might play a *cis*-acting regulatory role in transcription of *STAT3* and thus regulate the function of GCs in mammals. In this study, using porcine GCs as a model, the molecular mechanism regarding the regulation between *C/EBPβ* and *STAT3* was first identified, and then its biological functions were explored for cell apoptosis and proliferation.

## 2. Materials and Methods

### 2.1. Ethics Approval

All experiments in the present study were performed in accordance with the guidelines of the Animal Care and Use Committee of South China Agricultural University Guangzhou, China (approval number: SCAU#2013-10).

### 2.2. Prediction of Putative C/EBPβ Binding Sites on the Promoter of STAT3

**Figure 1:**
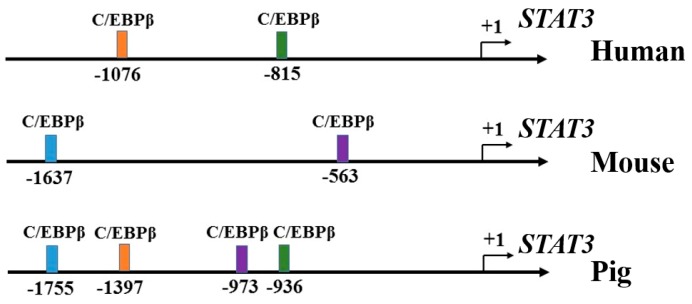
Putative binding sites of CCAAT/enhancer binding protein beta (*C/EBPβ)* on the promoter of Signal transducer and activator of transcription 3 *(STAT3)* in humans, mice, and pigs.

The promoter sequences of *STAT3* (upstream 2 kb) were download from NCBI for human [22], mouse [23] and pig [24]. TFBIND [25], Biobase [26], Jaspar [27] and Research [28] were used to predict the putative binding location of *C/EBPβ*. The putative binding sites of *C/EBPβ* concurrently predicted by all of those four tools were used for further analysis. The putative binding sites of *C/EBPβ* on the promoter of *STAT3* in humans, mice, and pigs are shown in Figure 1.

### 2.3. Construction of STAT3 5′ Deletion and Luciferase Assay

The genomic DNA of porcine ovary tissues was used as a template. PCR was performed using PrimerSTAR^®^ (TaKaRa, Dalian, Liaoning, China) high fidelity enzyme to obtain the *STAT3* promoter of 2575 bp. Primers are presented in Table 1. Then PCR products were purified by gelatinization and the addition of “A” tail to combine with pMD-18T, which were transformed into competent cells DH5α, inoculated on ampicillin-containing lysogeny broth (LB) plates at 37 °C for overnight. Further, monoclonal bacteria was added from platelets to ampicillin of LB medium, and incubated overnight at 37 °C shaker. The bacteria were collected by centrifugation and the plasmids were extracted. The correct plasmid for sequencing was named T-*STAT3*. Then we used T-*STAT3* as a template and designed five different upstream primers to amplify deletion fragments. The longest deletion fragment was named P1. The location of each deletion fragment of *STAT3* was P1 (−2199/+375), P2 (−1532/+375), P3 (−1035/+375), P4 (−587/+375) and P5 (−167/+375). We used the same method to obtain plasmids of each deletion fragment containing *MIu*I and *Xho*I cleavage sites (Table 1). Simultaneously, we cloned each deletion fragment into the eukaryotic expression vector pGL3-Basic, which digested with *MIu*I and *Xho*I restriction endonuclease. According to Promega’s dual luciferase reporter assay kit (Promega, Madison, WI, USA) and previous study [29], we use the BioTek Synergy 2 multifunctional microplate reader (BioTek, Winooski, VT, USA) for fluorescence detection. The ratio of the expression of firefly luciferase to renilla luciferase was the target fragment activity.

### 2.4. Culture of Porcine GCs In Vitro

Porcine ovarian GCs were cultured as previously described [30]. Briefly, porcine ovaries from prepubertal gilts were collected from a local slaughterhouse in Guangzhou, and transferred to our laboratory in phosphate-buffered saline (PBS) containing penicillin (100 IU/mL) and streptomycin (100 μg/mL) (Invitrogen, Shanghai, China) at a storage temperature of >30 °C. Subsequently, 3–5 mm follicles were punctured for GC collection using a 1-mL syringe, and the isolated GCs were washed twice with PBS preheated to 37 °C. The cells were seeded into 25-cm^2^ flasks and cultured at 37 °C under 5% CO_2_ in DMEM (Hyclone, Logan, UT, USA) containing 10% fetal bovine serum (Hyclone, Logan, UT, USA), 100 IU/mL penicillin, and 100 μg/mL streptomycin.

### 2.5. Chromatin Immunoprecipitation Assay

GCs were cross-linked until the cell density reached 70%. Then we discarded the original culture medium in the flask and the cells were sequentially treated with formaldehyde, glycine, PBS-Halt Cocktail and centrifuged to collect the cell pellet. The chromatin immunoprecipitation (ChIP) assay (Thermofisher, Rockford, IL, USA) was carried out according to the previous ChIP protocol [31]. ChIP primer for −1397/−1387 of *STAT3* is presented in Table 1 and was used to detect the binding of STAT3 and H3. After immunoprecipitation, the C/EBPβ binding site was identified by PCR amplification. Total fragmented DNA was used as input. DNA Marker was 100 bp.

### 2.6. Real-Time Quantitative PCR Analysis

At least three wells per group were collected for extraction of total RNA. Total RNA was extracted using TRIzol reagent (TaKaRa, Tokyo, Japan) and then reverse-transcribed using a RevertAid First Strand cDNA Synthesis Kit (Thermo Scientific, Waltham, MA, USA) for mRNAs. The relative expression levels of mRNAs were quantified using Maxima SYBR Green qRT-PCR Master Mix (2×) (Thermo Scientific, Waltham, MA, USA) and THUNDERBIRD SYBR qPCR Mix (Toyobo, Osaka, Japan) in a LightCycler Real-Time PCR system. The expression levels of *GAPDH* mRNAs were used as endogenous controls, and the fold changes of *STAT3* and *C/EBPβ* were calculated using the 2^−ΔΔct^ method. The primer sequences are listed in Table 1.

### 2.7. Cell Proliferation and Apoptosis Assay

Cell proliferation assays were performed using Cell-Light Edu Apollo 567 In Vitro Kit (RiboBio Co., Ltd., Guangzhou, Guangdong, China). GCs were seeded into 48-well plates at one day prior to transfection. When the cells reached 30–50% coverage of one well, pcDNA3.1-C/EBPβ, pcDNA3.1-STAT3, pcDNA3.1-Control, C/EBPβ-siRNA, STAT3-siRNA or siRNA-NC were transfected into the cells at different final concentrations for 48 h. The specific steps are: the Edu solution was diluted 1:1000 with cell culture media to prepare 50 μM Edu medium, add 100 μL of 50 μM Edu media to each well for 2 h, discard the culture medium, add 100 μL of cell fixing solution (80% acetone) to each well for 30 min at room temperature, wash twice with PBS, add 100 μL of penetrant (0.5% TritonX-100 in PBS) to permeabilize the cells and rinse once with PBS, add 100 μL of 1 × Apollo Staining Solution and incubate for 30 min at room temperature in the dark, discard staining solution and add 100 μL DAPI per well incubate for 30 min at room temperature in the dark, then add PBS to take pictures under microscope.

Cell apoptosis assays were performed using an Annexin V-FITC Apoptosis Detection Kit (BioVision, Milpitas, CA, USA) according to the manufacturer’s instructions. Briefly, GCs (1–5 × 10^5^ cells/well) were cultured in triplicate in 6-well plates at one day prior to transfection. When the cells reached 30–50% coverage of one well, pcDNA3.1-C/EBPβ, pcDNA3.1-STAT3, pcDNA3.1-Control, C/EBPβ-siRNA, STAT3-siRNA or siRNA-NC were transfected into the cells at different final concentrations for 48 h. The cells were then harvested, washed twice with ice-cold PBS, and resuspended in 500 μL of binding buffer. Next, 1.25 μL of Annexin V-FITC was added in the dark for 15 min at room temperature, then 1000× *g* centrifugation for 5 min at room temperature to remove the supernatant. The cells were gently resuspended with 0.5 mL precooling 1 × solution, and 10 μL of PI (propidium iodide; 50 μg/mL) were added. Last, the cells were analyzed in a flow cytometer (Becton Dickinson Co., San Jose, CA, USA) using the FITC signal detector (FL1) and phycoerythrin emission signal detector (FL2). All experiments were performed at least three times. Cells in the lower right quadrant are annexin-positive/PI-negative early apoptotic cells. The cells in the upper right quadrant are annexin-positive/PI-positive late apoptotic cells.

### 2.8. Western Blot Analysis

The cells were harvested and analyzed for their expression levels of total *STAT3* using an anti-STAT3 antibody (Santa Cruz Biotechnology, Santa Cruz, CA, USA). The molecular weight of STAT3 was 91 kDa. Protein concentrations were determined using a BCA Protein Assay Kit (Vigorous Bio-technology Beijing Co., Ltd., Beijing, China), and equal amounts of protein were separated by SDS-PAGE and electroblotted onto polyvinylidene difluoride membranes. The membranes were blocked with 5% nonfat milk in PBS containing a percentage of Tween-20 for 1 h, and then incubated with a primary antibody against hamartin (1:1000; Biorbyt, San Francisco, CA, USA) overnight at 4 °C. An anti-GAPDH antibody (1:3000; Sigma, St. Louis, MO, USA) was used as an internal control. After incubation with secondary antibodies for 1 h at room temperature, antibody-bound protein bands were visualized using an ECL-PLUS Kit (Amersham Biosciences, Piscataway, NJ, USA). The gray scale values of the bands were calculated using ImageJ software, which was free downloaded from NIH. The relative protein expression level of STAT3 was normalized by β-Actin values. At least three replicates were conducted for each group.

### 2.9. Data Analysis

All experiments were repeated at least three times independently. Data were expressed as means ± standard deviation (SD) of repeated experiments. Statistical analyses were carried out using R software. The significance of differences in means between two groups was analyzed by using Student’s *t*-test (two-tailed). * indicates *p* < 0.05; ** indicates *p* < 0.01.

## 3. Results

### 3.1. C/EBPβ Promotes the mRNA and Protein Level of STAT3

**Figure 2:**
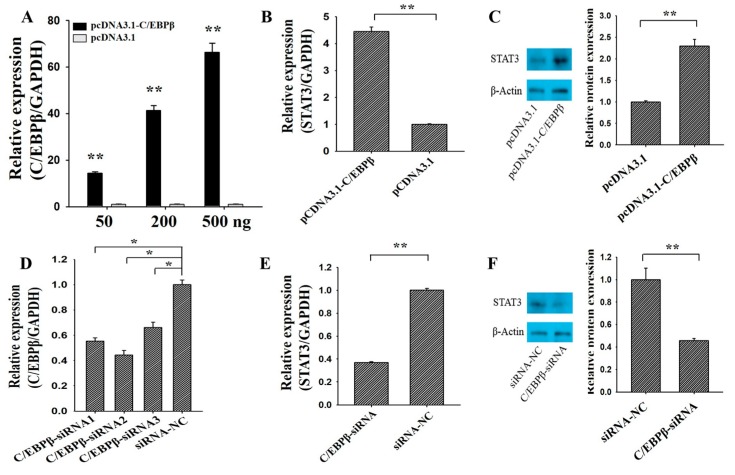
*C/EBPβ* promotes the mRNA and protein levels of *STAT3*. (**A**) Relative expression of *C/EBPβ* against the different concentrations of pcDNA3.1-C/EBPβ plasmid; the mRNA (**B**) and protein (**C**) expression of *STAT3* was stimulated by pcDNA3.1-*C/EBPβ*; (**D**) relative expression of *C/EBPβ* knockdown by three siRNAs; the mRNA (**E**) and protein (**F**) expression of *STAT3* depressed by C/EBPβ-siRNA. ** indicates *p* < 0.01; * indicates *p* < 0.05. Data were represented as means ± SD. siRNA: small interfering RNA; siRNA-NC: a siRNA negative control.

The overexpression plasmid and small interfering RNA (siRNA) of *C/EBPβ* were first built to explore the effects of *C/EBPβ* on the expression of *STAT3* (Figure 2). We found that the mRNA expression of *C/EBPβ* was increasing along with the concentration of pcDNA3.1-C/EBPβ (Figure 2A), and the 200 ng of pcDNA3.1-C/EBPβ plasmid was selected and used for further analysis. Compared to the control group, overexpression of C/EBPβ significantly up-regulated the mRNA (Figure 2B, *p* < 0.01) and protein (Figure 2C, *p* < 0.05) levels of STAT3. Three C/EBPβ-specific small interfering RNA (siRNA) (C/EBPβ-siRNA1, C/EBPβ-siRNA2 and C/EBPβ-siRNA3) and a negative control (siRNA-NC) were transfected into GCs to evaluate the inhibition efficiency for C/EBPβ (Figure 2D). As shown in Figure 2D, C/EBPβ-siRNA2 exhibited the best inhibition efficiency, and thus C/EBPβ-siRNA2 was selected for the knockdown of *C/EBPβ* in GCs. Compared with the control group, interfering with the expression of *C/EBPβ* significantly down-regulated the mRNA (Figure 2E, *p* < 0.01) and protein (Figure 2F, *p* < 0.05) levels of *STAT3*. These observations indicated that *C/EBPβ* could positively regulate the mRNA and protein amounts of *STAT3* in porcine GCs.

### 3.2. C/EBPβ Binding at −1397/−1387 Region of STAT3

**Figure 3:**
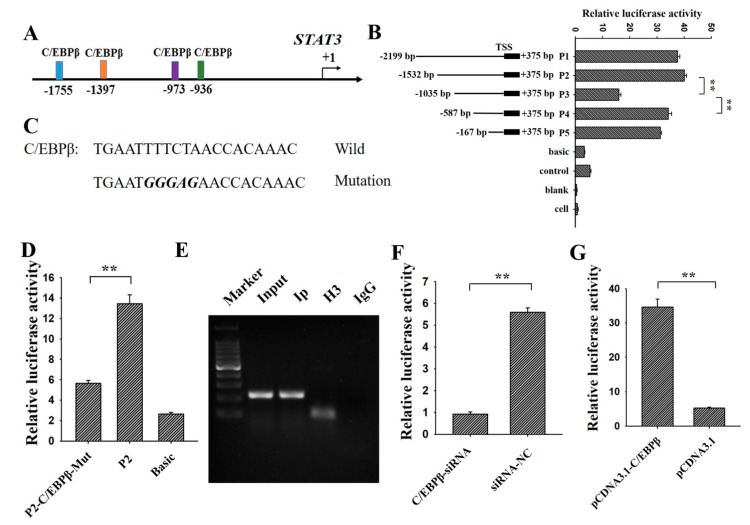
*C/EBPβ* binding at −1397/−1387 region of *STAT3*. (**A**) Predictions of potential *C/EBPβ* binding sites at the promoter of *STAT3*; (**B**) relative luciferase activity of *STAT3* promoter after the 5′ deletion of the potential *C/EBPβ* binding sites; (**C**) mutation of the potential *C/EBPβ* binding site (−1397/−1387); (**D**) relative luciferase activity of P2 after the mutation of −1397/−1387; (**E**) further confirmation of *C/EBPβ* binding at −1397/−1387 by ChIP. DNA Marker was 100 bp. Relative luciferase activity of P2 after the knockdown and over-expression of *C/EBPβ* by *C/EBPβ*-siRNA (**F**) and pcDNA3.1-*C/EBPβ* (**G**), respectively. ** indicates *p* < 0.01; * indicates *p* < 0.05. Data were represented as means ± SD.

Four putative binding sites of *C/EBPβ* were predicted on the promoter of *STAT3* in pigs (Figure 3A), suggesting *STAT3* might be a direct target of *C/EBPβ*. To investigate the molecular mechanism of C/EBPβ regulating the expression of *STAT3*, the 5′-deletions and gene reporter assay were constructed for *STAT3* (Figure 3B). Compared with P1, the relative luciferase activity of P2 did not showed significant changes with the deletion of the first putative binding site (−1755/−1747). Deletion of the second potential binding site (−1397/−1387) significantly reduced the relative luciferase activity of P2 (Figure 3B), which is accordance with the results of Figure 2. However, compared with P3, deletion of the third (−973/−964) and forth binding site (−936/−925) could significantly increase the relative luciferase activity (Figure 3B), which didn’t correspond with the results of Figure 2. Therefore, −1397/−1387 might be the exact binding site of *C/EBPβ* and was selected for further analysis.

To validate *STAT3* as a target of *C/EBPβ*, this potential site of P2 was mutated and then were cloned into the dual-luciferase reporter (Figure 3C). We found that mutation significantly reduced the relative luciferase activity of P2 (Figure 3D). Moreover, ChIP further confirmed that *C/EBPβ* bound at (−1397/−1387) in porcine GCs (Figure 3E), and siRNA-C/EBPβ significantly decreased the relative luciferase of P2 (Figure 3F, *p* < 0.01). Moreover, pcDNA3.1-C/EBPβ significantly increased the relative luciferase of P2 (Figure 3G, *p* < 0.01). These results suggested that *C/EBPβ* directly bound at −1397/−1387 of *STAT3* to positively regulate the transcription of *STAT3* in porcine GCs.

### 3.3. STAT3 Inhibits Apoptosis and Promotes Proliferation of Ovarian GCs

**Figure 4:**
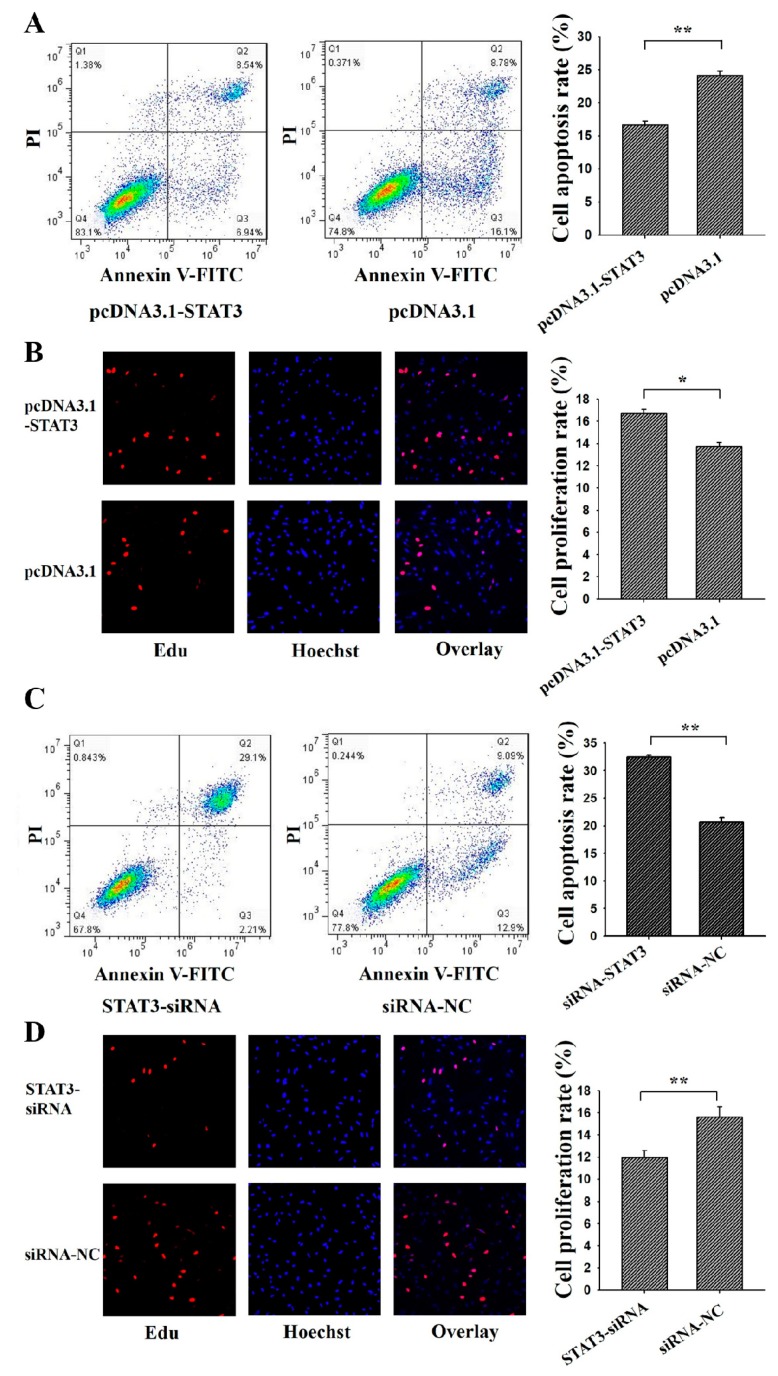
*STAT3* regulates cell apoptosis and proliferation of ovarian granulosa cells (GCs). PcDNA3.1-*STAT3* decreased cell apoptosis (**A**) and increased cell proliferation (**B**) in GCs as assessed by Annexin V-FITC/PI and Edu staining, respectively. SiRNA-*STAT3* increased cell apoptosis (**C**,**D**) decreased cell proliferation in porcine GCs as assessed by Annexin V-FITC/PI and Edu staining, respectively. For Annexin V-FITC/P analysis, cells in the lower right quadrant were annexin-positive/PI-negative early apoptotic cells. The cells in the upper right quadrant were annexin-positive/PI-positive late apoptotic cells. The percentage of cells undergoing early and late apoptosis were presented in the relating barplot. ** indicates *p* < 0.01; * indicates *p* < 0.05. Data were represented as means ± SD.

To determine the cellular function of *STAT3* on the apoptosis and proliferation, pcDNA3.1-*STAT3* or *STAT3*-siRNA was transfected into porcine GCs (Figure 4). Annexin V-FITC flow cytometry and Edu staining were used to analysis the cell apoptosis and proliferation, respectively. Results showed that the apoptosis rate of GCs in the pcDNA3.1-STAT3 group was significantly lower than the control group (Figure 4A), and the proliferation rate in the pcDNA3.1-STAT3 group was significantly higher than that in the control group (Figure 4B). Moreover, the apoptosis rate of GCs in *STAT3*-siRNA was significantly higher than that in the control group (Figure 4C), and the proliferation rate in STAT3-siRNA group was significantly lower than that in the control group (Figure 4D). These results suggested that *STAT3* might inhibit apoptosis and promote proliferation of ovarian GCs.

### 3.4. C/EBPβ Inhibits Apoptosis and Promotes Proliferation of Ovarian GCs

**Figure 5:**
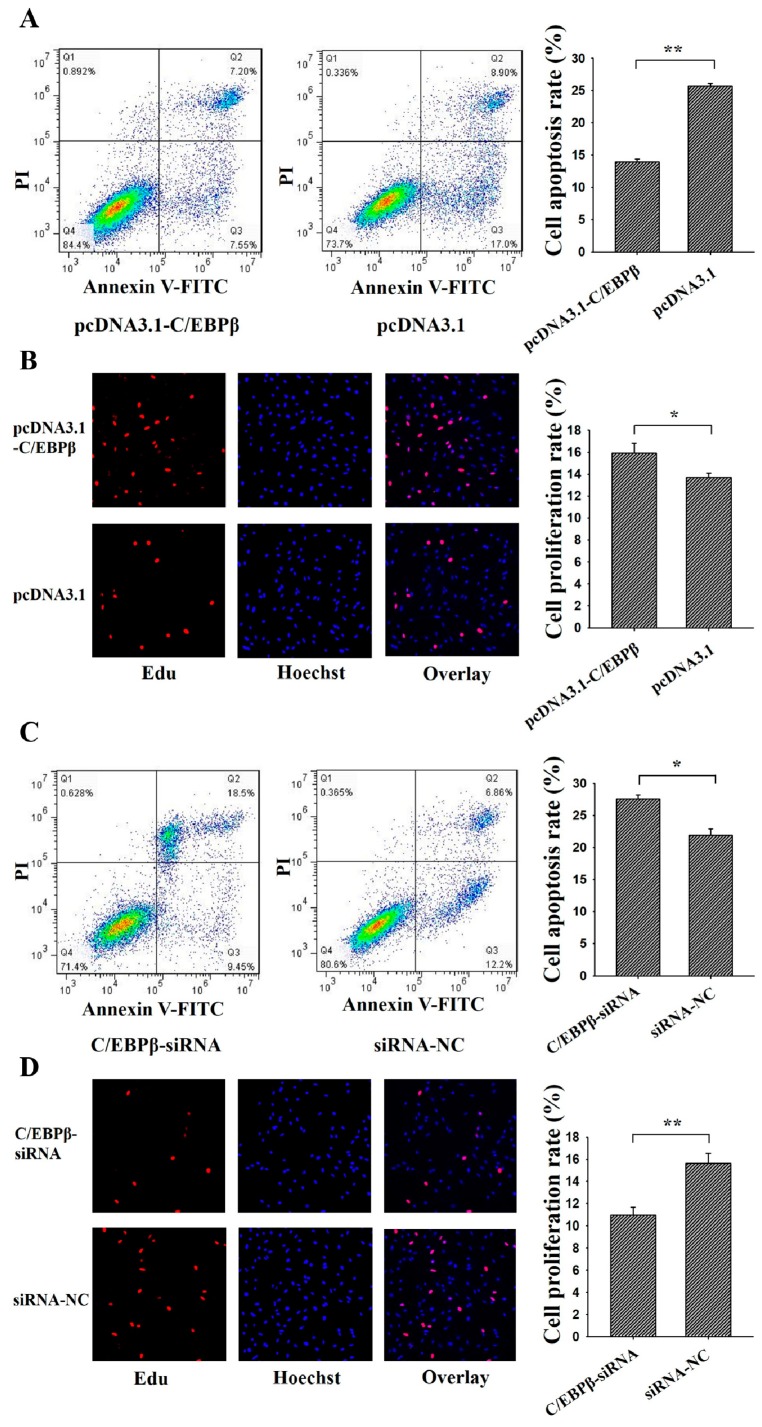
*C/EBPβ* regulates the apoptosis and proliferation of ovarian GCs. PcDNA3.1-*C/EBPβ* decreased cell apoptosis (**A**) and increased cell proliferation (**B**) in granulosa cells as assessed by Annexin V-FITC/PI and Edu staining, respectively. SiRNA-*C/EBPβ* increased cell apoptosis (**C**,**D**) decreased cell proliferation in granulosa cells as assessed by Annexin V-FITC/PI and Edu staining, respectively. For Annexin V-FITC/P analysis, cells in the lower right quadrant were annexin-positive/PI-negative early apoptotic cells. The cells in the upper right quadrant were annexin-positive/PI-positive late apoptotic cells. The percentage of cells undergoing early and late apoptosis were presented in the relating barplot. ** indicates *p* < 0.01; * indicates *p* < 0.05. Data were represented as means ± SD.

To investigate the function of *C/EBPβ* on cell apoptosis and cell proliferation, pcDNA3.1-*C/EBPβ* or *C/EBPβ*-siRNA was transfected into porcine GCs (Figure 5). Results showed that the apoptosis rate of GCs in pcDNA3.1-*C/EBPβ* group was significantly lower than that in the control group (Figure 5A), and the proliferation rate in the pcDNA3.1-*C/EBPβ* group was significantly higher than that in the control group (Figure 5B). Furthermore, the apoptosis rate of GCs in the *C/EBPβ*-siRNA group was significantly higher than that in the control group (Figure 5C), and the proliferation rate in the *C/EBPβ*-siRNA group was significantly lower than that in the control group (Figure 5D). These results demonstrated that *C/EBPβ* might inhibit cell apoptosis and promote cell proliferation for porcine GCs.

### 3.5. C/EBPβ Enhanced the Antiapoptotic and Pro-Proliferation Effects of STAT3 in Ovarian GCs

**Figure 6:**
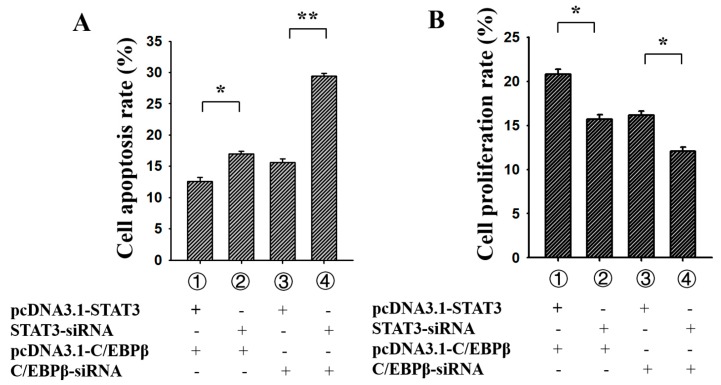
*C/EBPβ* enhanced the antiapoptotic and pro-proliferation effects of *STAT3* in porcine GCs. (**A**) *C/EBPβ* enhanced the antiapoptotic effects of *STAT3*; (**B**) *C/EBPβ* enhanced the pro-proliferation effects of *STAT3*. The percentage of cells undergoing early and late apoptosis were presented. ** indicates *p* < 0.01; * indicates *p* < 0.05. Data were represented as means ± SD.

PcDNA3.1-*C/EBPβ*, *C/EBPβ*-siRNA, pcDNA3.1-*STAT3* and *STAT3*-siRNA were co-transfected into porcine GCs to analyze the effect of *C/EBPβ* on the function of *STAT3*, respectively (Figure 6). For the apoptosis rate of porcine GCs (Figure 6A), group 1 (pcDNA3.1-*C/EBPβ* + pcDNA3.1-STAT3) was significantly lower than group 2 (pcDNA3.1-C/EBPβ + *STAT3*-siRNA), and group 3 (pcDNA3.1-*STAT3* + *C/EBPβ*-siRNA) was significantly lower than group 4 (*STAT3*-siRNA + *C/EBPβ*-siRNA). These results indicated that *C/EBPβ* might enhance the antiapoptotic effect of *STAT3* in ovarian GCs. For the proliferation rate of porcine GCs (Figure 6B), group 1 (pcDNA3.1-*C/EBPβ* + pcDNA3.1-*STAT3*) was significantly higher than group 2 (pcDNA3.1-*C/EBPβ* + *STAT3*-siRNA), while group 3 (pcDNA3.1-*STAT3* + *C/EBPβ*-siRNA) was significantly higher than group 4 (*STAT3*-siRNA + *C/EBPβ*-siRNA). These results suggested that *C/EBPβ* might enhance pro-proliferation effects of *STAT3* in ovarian GCs.

## 4. Discussion

It is widely known that the regular cell apoptosis and proliferation of GCs supported the development of follicles in mammals [5,6]. In mice, GC-specific *STAT3* knockout could impair the fertility with a significant reduction in litters and litter sizes [18], but oocyte-specific *STAT3* knockout had no effect on fertility [18]. Moreover, the GC-specific *C/EBPβ* knockout also resulted in the subfertility with a reduction of corpus luteums in 70% [19]. These results suggested that *STAT3* and *C/EBPβ* had exhibited an essential role in folliculogenesis and female reproduction. Previous studies recommended that *C/EBPβ* might display a cis-regulatory role in mammalian GCs [21,32]. We previously found there were several potential C/EBPβ sites on the promoter of *STAT3* in humans, mice, and pigs (Figure 1), and furthermore, we found that mRNA expression of *STAT3* had significantly increased or decreased along with the overexpression or depression of *C/EBPβ* (Figure 2). This observation indicated that *C/EBPβ* was likely to play a positive regulatory role for *STAT3* in porcine GCs.

In pigs, four putative *C/EBPβ* binding sites were predicted at the promoter of *STAT3* (Figure 3A), suggesting that *STAT3* might be a direct target of *C/EBPβ*. Based on the dual luciferase reporter system, the deletion of −1397/−1387 binding sites resulted in a significant reduction of the relative luciferase activity. This observation was in accordance with results shown in Figure 2. Furthermore, after mutation of this putative binding site, the relative luciferase activity showed a significant reduction compared to that of P2 (Figure 3C,D). Then *C/EBPβ* was further confirmed to bind at −1397/−1387 of *STAT3* by ChIP (Figure 3E). These resulted demonstrated that *C/EBPβ* directly targeted at *STAT3* to positively regulate the expression of *STAT3* in porcine GCs.

Previous studies have found that lower levels of phosphorylated *STAT3* in GCs may be related to ovarian leptin resistance and decreased fertilization in polycystic ovarian syndrome women [33]. *STAT3* is required to regulate the expression of luteinizting hormone receptor mRNA during the cell differentiation of GCs in mice [34]. In bovine GCs, protein expression of *STAT3* appears to be stronger in the ovulatory follicles as compared to the small follicles and dominant follicles, and these results mean that *STAT3* in GCs may be associated with the follicular growth [35], and phosphorylated STAT3 levels are dramatically higher in GCs of subordinate follicles than those in dominant follicles in bovines [36]. Furthermore, it is recently proposed that the stronger amount of pSTAT3 in small follicles is associated with GCs death and follicular atresia [37]. These observations suggested that *STAT3* got involved in the functions of GCs and follicular development. Most importantly, in human GCs, promoting ovarian GCs apoptosis and affecting the steroidogenesis could suppress the activation of *STAT3* [38], and reduced expression of *STAT3* could enhance the early apoptosis rate of mouse GCs [17]. These results indicated *STAT3* might induce the apoptosis of mammalian GCs. In the present study, we found that *STAT3* inhibited cell apoptosis and promotes cell proliferation in porcine GCs (Figure 4), and these results further identified the biological functions of *STAT3* on cell apoptosis and cell proliferation in mammals. 

*C/EBPβ* is essential for ovulation and luteinization [39,40]. Previous studies report that C/EBPα and *C/EBPβ* double-mutant could change expressions of *Prlr*, *Abcb1b1*, *Plxnd1* and *Cyp19a1* in mouse GCs, and moreover, putative *C/EBPβ* binding sites have been identified in the promoters of these genes [39]. *C/EBPβ* knockdown could alter *Star*, *Cyp11a1*, and *Hsd3b2* expressions in granulosa tumour-derived KGN cells [41] and result in a reduction of *Runx2* mRNA levels in periovulatory GC of mice and thus might impact cell differentiation of GCs to become luteal cells [42]. These observations suggested *C/EBPβ* was an important transcript factor in mammalian GCs. Moreover, by binding at the promoter, *C/EBPβ* was likely to be essentially required for Star, which was a vital accessory protein required for biosynthesis of steroid hormones from cholesterol, in response to hormones in rat [32], mouse [43], human [44], and porcine [45] GCs. In the present study, we found that *C/EBPβ* inhibited cell apoptosis and promoted cell proliferation in porcine GCs (Figure 5). We also found *C/EBPβ* enhanced the antiapoptotic and pro-proliferation effects of *STAT3* in porcine GCs (Figure 6). These results further certified and confirmed that *STAT3* was a target of C/EBPβ in mammalian GCs. Collectively, in porcine ovarian GCs, *C/EBPβ* targeted at −1397/−1387 region to promote the expression of *STAT3*, and moreover, *C/EBPβ* might enhance the antiapoptotic and pro-proliferative effects of *STAT3*. These present works could provide usefully biological information for further studies on the molecular mechanism of *C/EBPβ* and *STAT3* in ovarian follicular development.

## Figures and Tables

**Table 1:** Primers used in the present study.

Name	Sequence *	Product (bp)	Accession Number
P1 (−2199/+375)	F: CGACGCGT TCCTCAACCCACCAAGAAAG	2575	NM_001044580
	R: CCCTCGAG CTCCCGGTCTCTTCGTATCC		
P2 (−1532/+375)	F: CGACGCGT CTCCAAGTCATTGATTTTCT	1908	NM_001044580
	R: ditto		
P3 (−1035/+375)	F: CGACGCGT TACTAAACAAACACAATAAA	1410	NM_001044580
	R: ditto		
P4 (−587/+375)	F: CGACGCGT CTGAGGTTCAAAGCAGGCGG	963	NM_001044580
	R: ditto		
P5 (−167/+375)	F: CGACGCGT CTCTCCTCATTGGCAAGTGG	543	NM_001044580
	R: ditto		
qRT-PCR-STAT3	F: GGGCTTTATCAGTAAGGAGA R: GGAATGTCAGGGTAGAGGTA	276	NM_001044580
qRT-PCR -C/EBPβ	F: CGGACTGCAAGCGGAAGGAGGA R: GGCTGGACGACGAGGATGTGGA	153	NM_001199889
qRT-PCR-GAPDH	F: TCCCGCCAACATCAAAT R: CACGCCCATCACAAACAT	201	NM_001206359
ChIP-STAT3	F: ATAGCTATCCTTGGGGAGG R: AAGGGCCTGTTATCTCAC	150	NM_001044580

* The underlined is enzyme-cutting sites. The gray part is base protection.
